# Fragment size of lateral Hoffa fractures determines screw fixation trajectory: a human cadaveric cohort study

**DOI:** 10.2340/17453674.2024.40841

**Published:** 2024-06-14

**Authors:** Christian PEEZ, Ivan ZDERIC, Adrian DEICHSEL, Moritz LODDE, R Geoff RICHARDS, Boyko GUEORGUIEV, Christoph KITTL, Michael J RASCHKE, Elmar HERBST

**Affiliations:** 1 AO Research Institute Davos, Davos, Switzerland; 2 Department of Trauma, Hand and Reconstructive Surgery, University Hospital Münster, Münster, Germany

## Abstract

**Background and purpose:**

Recommendations regarding fragment-size-dependent screw fixation trajectory for coronal plane fractures of the posterior femoral condyles (Hoffa fractures) are lacking. The aim of this study was to compare the biomechanical properties of anteroposterior (AP) and crossed posteroanterior (PA) screw fixations across differently sized Hoffa fractures on human cadaveric femora.

**Patients and methods:**

4 different sizes of lateral Hoffa fractures (n = 12 x 4) were created in 48 distal human femora according to the Letenneur classification: (i) type I, (ii) type IIa, (ii) type IIb, and (iv) type IIc. Based on bone mineral density (BMD), specimens were assigned to the 4 fracture clusters and each cluster was further assigned to fixation with either AP (n = 6) or crossed PA screws (n = 6) to ensure homogeneity of BMD values and comparability between the different test conditions. All specimens were biomechanically tested under progressively increasing cyclic loading until failure, capturing the interfragmentary movements via motion tracking.

**Results:**

For Letenneur type I fractures, kilocycles to failure (mean difference [∆] 2.1, 95% confidence interval [CI] –1.3 to 5.5), failure load (∆ 105 N, CI –83 to 293), axial displacement (∆ 0.3 mm, CI –0.8 to 1.3), and fragment rotation (∆ 0.5°, CI –3.2 to 2.1) over 5.0 kilocycles did not differ significantly between the 2 screw trajectories. For each separate subtype of Letenneur type II fractures, fixation with crossed PA screws resulted in significantly higher kilocycles to failure (∆ 6.7, CI 3.3–10.1 to ∆ 8.9, CI 5.5–12.3) and failure load (∆ 275 N, CI 87–463 to ∆ 438, CI 250–626), as well as, less axial displacement from 3.0 kilocycles onwards (∆ 0.4°, CI 0.03–0.7 to ∆ 0.5°, CI 0.01–0.9) compared with AP screw fixation.

**Conclusion:**

Irrespective of the size of Letenneur type II fractures, crossed PA screw fixation provided greater biomechanical stability than AP-configured screws, whereas both screw fixation techniques demonstrated comparable biomechanical competence for Letenneur type I fractures. Fragment-size-dependent treatment strategies might be helpful to determine not only the screw configuration but also the surgical approach.

Intra-articular distal femur fractures represent rare but devastating injuries to the knee joint, accounting for up to 13% of all femur fractures [1]. These fractures frequently show coronal shear fragments of 1 or both posterior femoral condyles, known as Hoffa fractures. Despite modern surgical developments, the treatment of these intraarticular fracture patterns remains challenging as demonstrated in clinical studies reporting unsatisfactory functional outcomes [2-5]. Key factors to improve patient-reported outcomes and reduce the risk of post-traumatic osteoarthritis are anatomic reconstruction of the articular surface and preservation of the limb alignment, for which proper fracture reduction is essential [6,7].

To achieve absolute stability and interfragmentary compression, screw fixation currently represents the most common internal fixation method [2,8-10]. However, various fixation techniques have been proposed differing in size, number, configuration, and design of the screws [10-13]. In this context, the biomechanical stability of different screw configurations has been predominantly discussed for large Hoffa fragments (Letenneur type I and III) with conflicting results [11,12,14], whereas the more common Letenneur type II fractures have been consistently neglected [15]. However, these smaller Hoffa fracture fragments are more challenging to address with the commonly recommended indirect anteroposterior (AP) screws, as demonstrated by inconsistent and heterogeneous clinical results [2-5]. Therefore, direct fixation with crossed posteroanterior (PA) screws could be advantageous in such fracture patterns. However, there is a lack of evidence regarding optimal screw fixation trajectories in differently sized Hoffa fragments.

The aim of this study was to compare the biomechanical competence of AP and crossed PA screw fixations across differently sized Hoffa fractures. It was hypothesized that both screw trajectories would demonstrate comparable stability in large Hoffa fragments (Letenneur type I), whereas smaller Hoffa fragments (Letenneur type IIa–IIc) may require a direct crossed PA screw fixation.

## Methods

48 fresh-frozen (–20 °C) human cadaveric knees with a mean age of 68 years (standard deviation [SD] 10 years; 24 female and 24 donors) were obtained from an international tissue bank (Science Care, Phoenix, AZ, USA). The donors bequeathed their corpse for use in medical science during their lifetime. Written consent was obtained, so that no local or national ethical approval was required. The study is reported according to the STROBE (Strengthening the Reporting of Observational Studies in Epidemiology) guidelines.

The distal femora of all knees were assessed for bone mineral density (BMD) within the trabecular region of their femoral condyles using computed tomography (CT) scanning (Revolution EVO, General Electric Healthcare, Chalfont St Giles, UK) and subsequent image analysis (Amira, v.6.0, Thermo Fisher Scientific, Waltham, MA, USA) with segmentation between 150 and 450 mgHA/cm^3^.

**Figure 1:**
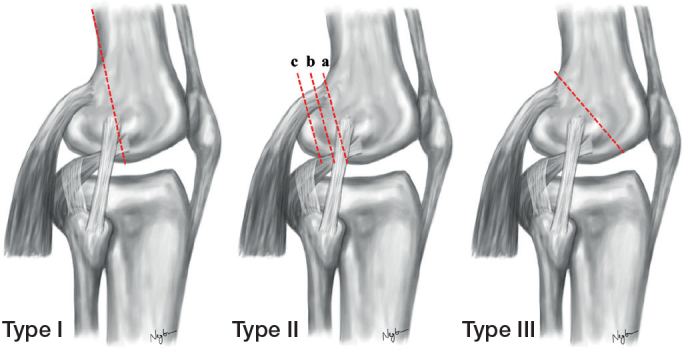
Schematic illustration of the Letenneur classification. Type I shows a fracture line through the lateral femoral condyle that is in line with the posterior cortex and involves 100% of the posterior femoral condyle. Type II are osteochondral fractures of the lateral femoral condyle with its subclassifications moving more posteriorly (IIa, IIb, and IIc—involving 75%, 50%, and 25% of the posterior femoral condyle). Type III depicts an oblique fracture line intersecting the articular surface more anteriorly.

Based on BMD, the knees were assigned to 4 clusters (n = 12) simulating lateral Hoffa fractures of different sizes according to the Letenneur classification [16]: (i) type I, (ii) type IIa, (iii) type IIb, and (iv) type IIc (Figure 1). The specimens of each cluster were assigned to 2 groups (n = 6) for AP or crossed PA screw fixation. Block randomization was used to allocate the specimens to the different testing conditions, such that the BMD values were homogeneously distributed between the 4 fracture clusters (mean difference [∆] –5.7 mgHA/cm^3^ to ∆ 10.8 mgHA/cm^3^) and the 2 fixation techniques within each fracture type (∆ –6.7 mgHA/cm^3^ to ∆ 4.2 mgHA/cm^3^). The allocation was based on BMD to ensure a high degree of standardization and thus comparability between the different testing conditions [17].

### Specimens preparation

**Figure 2:**
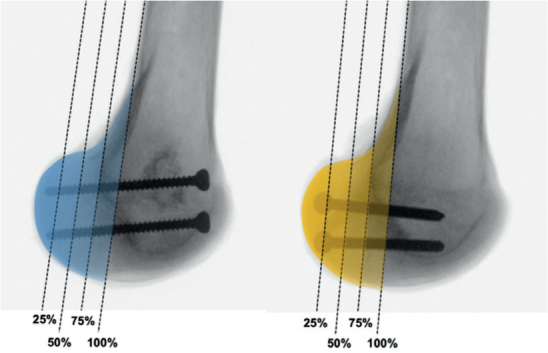
Mediolateral radiographs of exemplified specimens with anteroposterior (left) or crossed posteroanterior (right) screw fixation visualizing the different size-dependent modeling of lateral Hoffa fractures, according to the Letenneur classification. Letenneur type I, IIa, IIb, and IIc—involving 100%, 75%, 50%, and 25% of the posterior lateral femoral condyle, respectively.

After thawing the knees at room temperature for 24 hours, the distal femora were cut 250 mm proximal to the joint line. Once the knees were disarticulated, the entire soft tissue was removed to harvest the distal femora. Based on the cluster assignment, differently sized Hoffa fractures were simulated by setting an osteotomy in the coronal plane of the lateral posterior femoral condyle as previously described [12,14]. To mimic a Letenneur type I fracture involving 100% of the lateral posterior femoral condyle, the osteotomy plane was parallel to the axis of the posterior femoral cortex, such that the osteotomy extended from the extra-articular condyle–shaft junction distally to the articular surface [16]. To reproduce Letenneur type II fractures of different sizes, the AP diameter of the lateral femoral condyle was first determined from its posterior border to the tangent to the posterior femoral cortex. Then, an osteotomy was performed parallel to the posterior cortex according to a fragment size involving either 75%, 50%, or 25% of the lateral posterior femoral condyle, corresponding to a Letenneur type IIa, IIb, and IIc fracture, respectively [16] (Figure 2).

**Figure 3:**
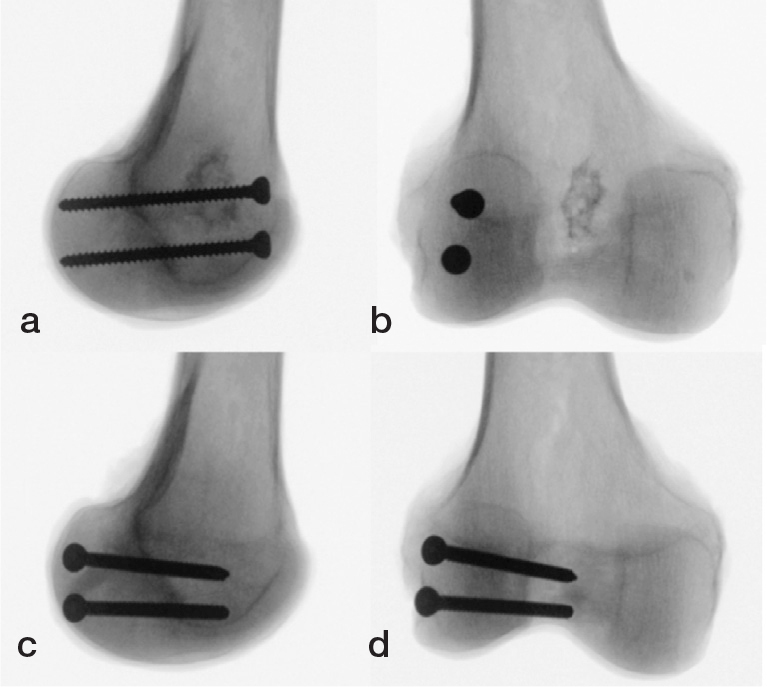
Mediolateral (a, c) and anteroposterior (b, d) radiographs of exemplified specimens with (a, b) anteroposterior and (c, d) crossed posteroanterior screw fixations.

Following anatomic fracture reduction, the fragments were fixed according to the specimen’s group assignment with use of either AP or crossed PA screws. For AP screw fixation, 2 parallel 4.5-mm fully threaded cortical screws (DePuy Synthes, Zuchwill, Switzerland) were inserted from the non-articular portion of the femoral trochlea and directed posteriorly across the fracture line (Figures 3a and 3b). For crossed PA screw fixation, 2 parallel 4.5-mm fully threaded cortical screws (same brand) were inserted from the non-articular lateral aspect of the condylar fragment, aiming from the coronal plane of femoral condyle at an inclination of 45° anteriorly and 10° distally (Figures 3c and 3d). Through this trajectory, the 2 parallel screws cross the central metaphysis of the distal femur and the tips are placed at the transition to the medial femoral condyle to prevent penetration into the patellofemoral joint as would be the case with strict PA screws [12].

Once the surgical procedures were performed, the proximal 6 cm of the femora were embedded in a polymethylmethacrylate (PMMA, Suter Kunststoffe AG, Fraubrunnen, Switzerland) socket. Finally, retro-reflective marker sets were attached to the femoral shaft and Hoffa fragment for motion tracking.

### Biomechanical testing

**Figure 4:**
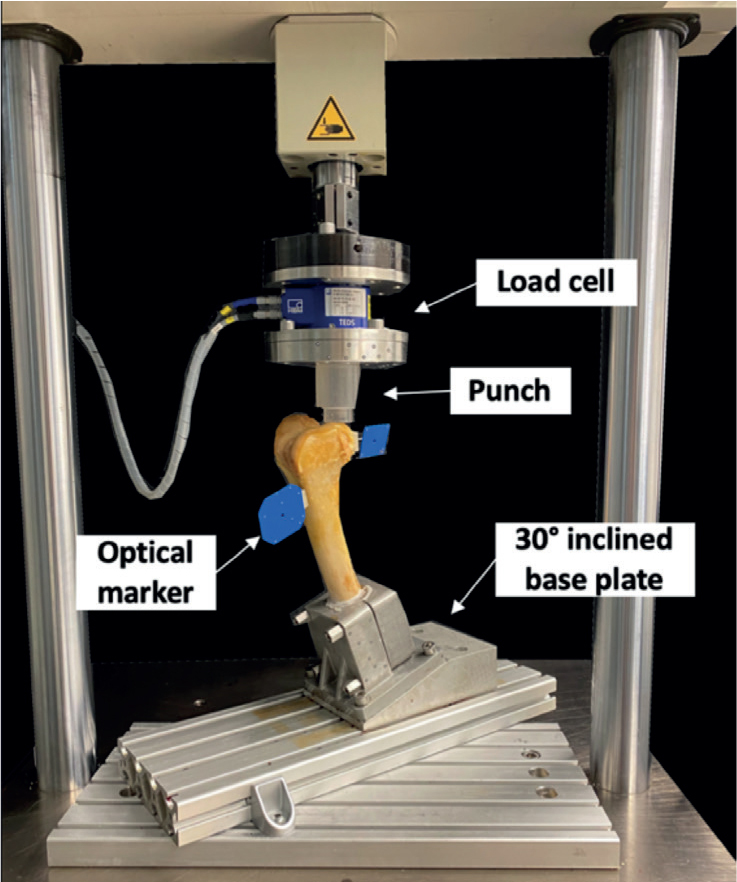
Setup with a specimen mounted for biomechanical testing.

Biomechanical testing was performed on a servo-hydraulic materials testing machine (Bionix 858.20, MTS Systems Corp, Eden Prairie, MN, USA) equipped with a 5 kN load cell (HBM, Darmstadt, Germany). Each specimen was tested in an inverted upright standing position. For this purpose, the femoral part of each femur was rigidly mounted to the machine base via an aluminum base plate, inclined at 30° in the sagittal plane to simulate axial loading of the Hoffa fragments in 30° knee flexion. Axial compression was applied via a custom-made PMMA punch allowing a homogenous load transfer to the hemispherical surface of the lateral posterior femoral condyle (Figure 4).

The loading protocol commenced with a non-destructive quasi-static ramp from 20 N preload to 100 N at a rate of 20 N/sec, followed by a progressively increasing cyclic loading at 2 Hz. While maintaining a constant valley load of 20 N, the peak load of each cycle increased monotonically from 100 N at a rate of 0.05 N/cycle until reaching 10 mm actuator displacement relative to the test start.

### Data acquisition and evaluation

Based on the actuator displacement and axial compression forces, force-displacement curves were generated to calculate construct stiffness, defined as the slope of the initial quasi-static ramp within the linear loading range between 20 and 100 N.

An optical motion tracking system (Aramis SRX, Carl Zeiss GOM Metrology GmbH, Braunschweig, Germany), operating at a maximum acceptance error of 0.004 mm [18], was used to capture the 3-dimensional coordinates of the markers and evaluate the interfragmentary movements in all 6 degrees of freedom at the initial stage and thereafter at 1.0, 2.0, 3.0, 4.0, and 5.0 kilocycles under peak loading conditions with respect to the beginning of the test. Specifically, fracture site displacement along the femoral shaft axis—defined as axial displacement—was captured at the most articular margin of the Hoffa fragment and the vertical osteotomy plane. Further, interfragmentary rotations of the lateral femoral condyle around the mediolateral axis—defined as fracture gap opening—and around the anteroposterior axis—defined as fracture gap twisting—were evaluated. As fracture step-offs greater than 2 mm are associated with an increased risk of osteoarthritis following tibial plateau fractures [19,20], reaching an axial displacement of 2.0 mm was set as a clinically relevant failure criterion for intra-articular distal femur fractures. The number of cycles until fulfillment of this criterion under peak loading—defined as cycles to failure—and the corresponding peak load—defined as failure load—were calculated.

### Statistics

Based on a previous study evaluating the biomechanical performance of different screw configurations for fixation of Hoffa fractures [12], an a priori power analysis was performed to detect a difference of 2.0 mm fracture displacement (effect size 0.9; power 0.8) using G*Power-2 software (University Düsseldorf, Düsseldorf, Germany) [21]. Based on this, a minimum sample size of 6 specimens per group was calculated.

Statistical analysis was performed using Prism (Version 9, GraphPad Software, Boston, MA, USA). Descriptive data is presented as mean value with SD, between-group differences as mean differences (∆) with 95% confidence intervals (CI). Normality of data distribution within each fracture model and fixation technique was tested and proved using the Shapiro–Wilk test, followed by a one-way analysis of variance (ANOVA) to confirm appropriate BMD-based randomization of the specimens. Two-way repeated-measures ANOVA with Geisser–Greenhouse correction were performed to compare the fixation strength in terms of cycles to failure, failure load, axial displacement, fracture gap twisting, and fracture gap opening (dependent variables) across the fragment size and fixation techniques (independent variables). Post-hoc Sidak testing was performed to account for multiple comparisons. The overall level of significance was set at 0.05.

### Ethics, registration, funding, and disclosures

Written consent was obtained, so that no local or national ethical approval was required. Due to the biomechanical nature of this research, registration in the German Clinical Trials Register was not required. All data is available from the corresponding author upon reasonable request. For this research, the authors did not receive funds, grants, or other support from any organization. All other authors declared no conflicts of interest. Complete disclosure of interest forms according to ICMJE are available on the article page, doi: 10.2340/17453674.2024.40841

## Results

BMD (mgHA/cm^3^) ranged on average from 135 (SD 37) to 147 (SD 36) for AP screw fixation and from 135 (SD 36) to 147 (SD 34) for PA screw fixation, demonstrating a homogeneous distribution among the groups within each fracture type with mean differences from –6.7 to 4.2 (Table).

**Table:** Mean values (standard deviation) of bone mineral density (BMD), kilocycles to failure, and failure load of the specimens with different types of Hoffa fractures following anteroposterior (AP) or crossed posteroanterior (PA) screw fixation including the mean differences between the 2 fixation techniques with 95% confidence interval (CI) and P value

Type	AP screw fixation	Crossed PA screw fixation	Mean difference (CI)	P value
Letenneur type I				
BMD (mgHA/cm^3^)	136 (37)	135 (36)	–0.7	
Kilocycles to failure	6.8 (2.8)	8.8 (4.1)	2.1 (–1.3 to 5.5)	0.4
Failure load (N)	434 (139)	539 (206)	106 (–83 to 293)	0.5
Letenneur type IIa				
BMD (mgHA/cm^3^)	142 (49)	146 (46)	4.2	
Kilocycles to failure	5.4 (1.8)	12.1 (5.0)	6.7 (3.3 to 10.1)	< 0.001
Failure load (N)	383 (107)	659 (319)	275 (87 to 463)	< 0.001
Letenneur type IIb				
BMD (mgHA/cm^3^)	135 (37)	142 (46)	–6.7	
Kilocycles to failure	6.1 (1.3)	14.7 (4.0)	8.6 (5.2 to 12.0)	< 0.001
Failure load (N)	406 (63)	836 (200)	430 (242 to 618)	< 0.001
Letenneur type IIc				
BMD (mgHA/cm^3^)	147 (36)	147 (34)	0.8	
Kilocycles to failure	5.4 (2.0)	14.3 (3.4)	8.9 (5.5 to 12.3)	< 0.001
Failure load (N)	368 (98)	806 (185)	438 (250 to 626)	< 0.001

### Letenneur type I fractures

**Figure 5:**
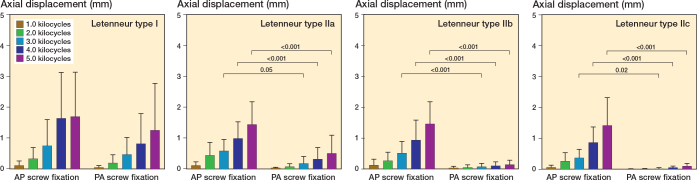
Axial displacement of the different types of Hoffa fractures following anteroposterior (AP) or crossed posteroanterior (PA) screw fixation, presented as mean value and standard deviation over the course of cyclic testing, together with the corresponding P value from the statistical comparisons between groups.

**Figure 6:**
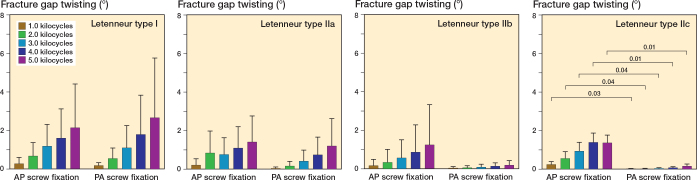
Fracture gap twisting of the different types of Hoffa fractures following anteroposterior (AP) or crossed posteroanterior (PA) screw fixation, presented as mean value and standard deviation over the course of cyclic testing, together with the corresponding P value from the statistical comparisons between groups.

**Figure 7:**
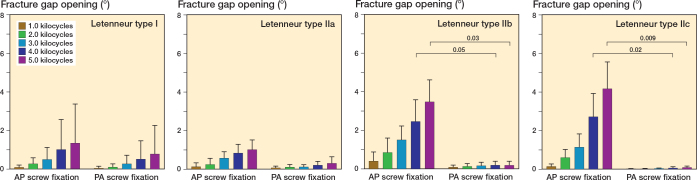
Fracture gap opening of the different types of Hoffa fractures following anteroposterior (AP) or crossed posteroanterior (PA) screw fixation, presented as mean value and standard deviation over the course of cyclic testing, together with the corresponding P value from the statistical comparisons between groups.

**Figure 8:**
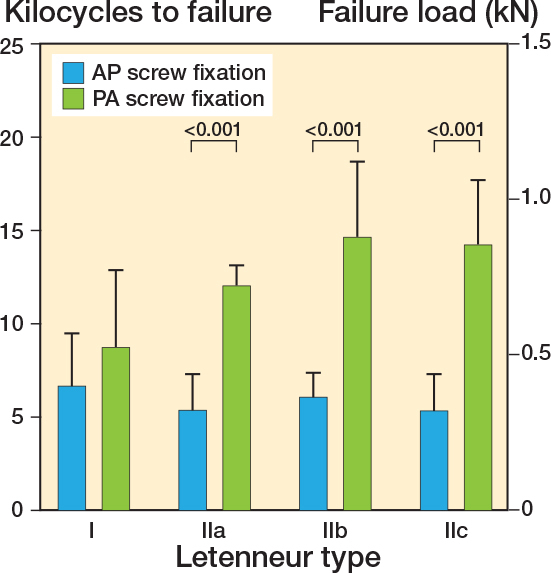
Kilocycles to failure and corresponding failure load (kN) of the different types of Hoffa fractures following anteroposterior (AP) or crossed posteroanterior (PA) screw fixation, presented as mean value and standard deviation, together with the corresponding P value from the statistical comparisons between groups.

Fixation of Letenneur type I fractures with either AP or crossed PA configurated screws resulted in comparable axial displacement (∆ 0.3 mm, CI –0.8 to 1.3), fracture gap twisting (∆ 0.5°, CI –3.2 to 2.1) and fracture gap opening (∆ 0.6°, CI –2.3 to 3.5) throughout all 5,000 test cycles (Figure 5–7). Both fixation techniques provided comparable numbers of kilocycles to failure (∆ 2.1, CI –1.3 to 5.5) and corresponding failure loads (∆ 105 N, CI –83 to 293) (Table and Figure 8).

### Letenneur type II fractures

In Letenneur type II fractures, crossed PA screw fixation exhibited significantly less axial displacement from 3.0 kilocycles onwards (∆ 0.4 mm, CI 0.03–0.7 to ∆ 0.5 mm, CI 0.01–0.9) compared with AP screw fixation, regardless of fragment size (Figure 5). After 1,000 loading cycles, fracture gap twisting was significantly higher for AP screw fixation than for crossed PA screw fixated Letenneur type IIc fractures (∆ 0.2°, CI 0.03–0.5), while no significant difference was observed for Letenneur type IIa and IIb fractures (∆ 0.4°, CI –1.1 to 2.0 and ∆ 1.1°, CI –2.4 to 4.5) (Figure 6). Complementary AP screw fixation of Letenneur type IIb and IIc fractures resulted in a significantly increased fracture gap opening from 4.0 kilocycles onwards compared with crossed PA screw fixation (∆ 2.3°, CI 0.2–4.4 and ∆ 4.5°, CI 1.6–7.3), while both fixation techniques demonstrated a comparable fracture gap opening in Letenneur type IIa fractures (∆ 0.7°, CI –1.1 to 2.5) (Figure 7). For each separate subtype of Letenneur II fractures, crossed PA screw configuration exhibited a significantly higher number of kilocycles to failure than AP screw fixation (∆ 6.7, CI 3.3–10.1 to ∆ 8.9, CI 5.5–12.3) and significantly higher corresponding failure loads (∆ 275 N, CI 87–463 to ∆ 438 N, CI 250–626) (Table and Figure 8).

## Discussion

We aimed to investigate the biomechanical properties of AP and crossed PA screw fixations across differently sized Hoffa fractures on human cadaveric femora.

The most important finding of our study was that the biomechanical stability of Hoffa fractures treated with screw fixation was strongly dependent on the fragment size and screw configuration. For all subtypes of Letenneur type II fractures, crossed PA screw fixation provided higher biomechanical stability than AP screw configuration, with less axial displacement from 3.0 kilocycles onwards as well as a higher number of cycles to failure and failure load. In contrast, both techniques for fixation of Letenneur type I fractures (with large posterior condylar fragment) using either AP or crossed PA configurated screws demonstrated comparable biomechanical performance.

In our study, the 2 most common screw configurations were investigated in differently sized Hoffa fractures to develop fragment-size-dependent treatment recommendations. In Hoffa fractures with large posterior condylar fragments (Letenneur type I), both AP and crossed PA screw fixations exhibited similar biomechanical stability with comparable failure loads and axial displacement. In these fractures, the stability of the screw osteosynthesis might be further increased when using larger screw diameters. In 2020, Yao et al. [12] showed in a synthetic bone model that both AP and crossed PA orientated fixations with 6.5-mm screws had twice as high failure loads versus our results.

Another way to optimize the fixation stability might be the use of strictly PA orientated screws. Applying a comparable loading protocol as in the current study, Jarit et al. [11] demonstrated in a cadaveric bone model that lag screw fixation of Letenneur type I fractures with strictly PA orientated screws provided even higher biomechanical stability with less displacement at 10,000 cycles (0.67 mm) and higher failure load (1.7 kN) compared with the results in the present study. However, the use of strictly PA orientated screws requires their placement through the articular cartilage of the posterior femoral condyle, thus bearing the risk of extensive iatrogenic damage to the weight-bearing articular surface. Therefore, strict PA screw osteosynthesis does not appear to be useful in everyday clinical practice, particularly when using large diameter screws as biomechanically recommended [12,22].

Lateral Hoffa fractures commonly show smaller fragments, frequently involving less than 50% of the posterior femoral condyle (Letenneur type IIb) [15]. The smaller the fragment size, the more challenging it is to fix the posterior condylar fragment with AP screws. In fact, several clinical studies have shown that indirect screw fixation of such small Hoffa fragments frequently resulted in unsatisfactory functional outcomes [2-5]. Our study has demonstrated that smaller Letenneur type II fractures should be addressed with crossed PA screws. For such small Hoffa fractures, fixation with crossed PA configurated screws exhibited higher failure loads and less axial displacement from 3.0 kilocycles onwards compared with AP screw fixation, which was independently shown for all Letenneur II subtypes. In comparison with Letenneur type I fractures, the decrease of the fragment size resulted in improved stability of the bone–implant construct following PA screw fixation, as indicated by an increase in both cycles to failure and failure load for Letenneur type II fractures. This effect might be due to the fact that the Letenneur classification is based on the AP diameter of the lateral femoral condyle. As the size of the Hoffa fragment decreases, like in smaller Letenneur type II fractures, the size of the rigid counterpart of the distal femoral epiphysis increases reciprocally [16]. Thus, smaller Hoffa fractures provide a favorable size ratio between the mobile fracture fragment and the relatively larger counterpart of the distal femoral epiphysis for fixation of the Hoffa fragment with the PA screws. In contrast, larger Hoffa fractures involving the entire posterior femoral condyle (Letenneur type I and III) have a balanced size ratio between the Hoffa fragment and the rigid counterpart for fixation, so that both AP and crossed PA screw fixation provide comparable biomechanical stability in Letenneur type I fractures. Therefore, from a biomechanical point of view, crossed PA screw fixation may be advantageous in the surgical treatment of Letenneur type II fractures.

The outcomes from our study are of clinical relevance because the size of the Hoffa fragments has recently been considered the key determinant for surgical approach selection [7,23]. Because both AP and crossed PA screw fixations provided comparable stability in Letenneur type I fractures, several studies recommended treating these large coronal plane fractures via a lateral parapatellar approach [2,5,13,24] or posterolateral approaches [25]. However, the lateral parapatellar approach requires a hyperflexed knee position for an optimal visualization of the articular surface and anatomic fracture reduction [7,23], although knee extension might facilitate the reduction due to ligamentotaxis by both anterior cruciate and lateral collateral ligaments. Additionally, this approach hinders placement of an augmenting plate osteosynthesis, whereas posterolateral approaches provide the necessary trajectory for crossed PA screws and also enable augmentation by a posterior buttress or lateral plates [14,26,27]. In contrast, smaller Hoffa fragments can only be sufficiently addressed with crossed PA screws, so that posterolateral approaches might be advantageous in the surgical treatment of Letenneur type II fractures [25]. However, posterolateral approaches bear the risk of iatrogenic damage to the common peroneal nerve and the posterolateral ligamentous structures. Nevertheless, the nerve can be protected by using a classic posterolateral approach between the biceps femoris tendon and the iliotibial band [25], which provides sufficient exposure of the fracture and allows extension of the approach via a posterolateral capsulotomy or an osteotomy of the lateral femoral condyle to visualize the articular surface [25] if the commonly observed comminution areas are present [15]. Therefore, knowledge of fragment size-dependent stability and surgical approach-specific trajectories for screw and plate osteosynthesis allows development of individualized surgical treatment strategies, which might potentially improve functional outcomes by reducing treatment failures.

### Limitations

The present study has several limitations. First, only lateral Hoffa fractures without comminution zones were analyzed, although central comminution areas are frequently observed in the clinical setting, especially in case of smaller Hoffa fractures (Letenneur type II). Nevertheless, the fracture model used is characterized by high reproducibility enabling reliable conclusions regarding the fragment-size-dependent stability of AP and crossed PA screw configurations. Second, the study was performed on human cadaveric specimens from donors older than the representative population of patients suffering from a Hoffa fracture [28]. Nonetheless, the distal femora of all knees were assessed for BMD to confirm the use of non-osteoporotic specimens. Lastly, the biomechanical performance of the 2 different screw configurations was only assessed in 30° knee flexion, although the posterior femoral condyles are exposed to higher forces in deeper knee flexion [29].

### Conclusion

Irrespective of the size of Letenneur type II fractures, crossed PA screw fixation provided greater biomechanical stability than AP-configurated screws, whereas both screw fixation techniques demonstrated comparable biomechanical competence for Letenneur type I fractures. Fragment-size-dependent treatment strategies might be helpful to determine not only the screw configuration but also the surgical approach to potentially improve functional outcomes by reducing treatment failures.

## Notes

CP: Conception and design, testing and data acquisition, statistical analysis, writing. EH: Conception and design of the study, writing, internal review. BG and IZ: Conception and design of the study, internal review. AD, CK, RGR, ML, and MJR: Internal review.

The authors thank Lesli von Negenborn for assisting with the illustrations presented in this study.

Handling co-editors: Ivan Hvid and Robin Christensen

*Acta* thanks Mats Brittberg and Petri Virolainen for help with peer review of this manuscript.
